# Supplementary material for: Soil Bacterial Community Structure in Turfy Swamp and Its Response to Highway Disturbance
Source: Int J Environ Res Public Health. 2020 Oct 26;17(21):7822. doi: 10.3390/ijerph17217822 (PMC7663615; doi:10.3390/ijerph17217822)
Supplement: Supplementary file 1 [file ijerph-17-07822-s001.pdf]

Soil bacterial community structure in turfy swamp and its response to highway disturbance

Yuanyuan He[1], Yan Xu[1], Yan Lv[1,*], Lei Nie[1] and Hong Wang[2]

[1]Construction Engineering College, Jilin University, Xi Min Zhu Street, Changchun, Jilin, China ,130026; hyy20@mails.jlu.edu.cn, xuyan8102@jlu.edu.cn, lvyy@jlu.edu.cn, nielei@ jlu.edu.cn

[2]Collge of Civil Engineering，GuiZhou University, Jiaxiu South Road, Huaxi District, Guiyang, Guizhou, China, 550025; wanghong15@mails.jlu.edu.cn.

*Correspondence: e-mail: lvyy@jlu.edu.cn

Table S1. Multiple linear regression analyses between OTU and the tested environmental variables.

Variables Entered/Removed[a]

Model	Variables Entered	Variables Removed	Method
1	WT	.	Stepwise (Criteria: Probability-of-F-to-enter ≤ 0.050; Probability-of-F-to-remove ≥ 0.100).

a. Dependent Variable: OTU

Model Summary[b]

Model	R	R Square	Adjusted R Square	Std. Error of the Estimate	Durbin-Watson
1	0.807[a]	0.652	0.630	214.952	1.232

a. Predictors: (Constant), WT: b. Dependent Variable: OTU

ANOVA[a]

Model		Sum of Squares	df	Mean Square	F	Sig.
1	Regression	1382514.327	1	1382514.327	29.922	0.000[b]
	Residual	739270.118	16	46204.382		
	Total	2121784.444	17			

a. Dependent Variable: OTU, b. Predictors: (Constant), WT.

Coefficients[a]

Model		Unstandardized Coefficients		Standardized Coefficients	t	Sig.	Collinearity Statistics	
		B	Std. Error	Beta			Tolerance	VIF
1	(Constant)	3153.134	111.562		28.264	0.000		
	WT	19.747	3.610	0.807	5.470	0.000	1.000	1.000

a. Dependent Variable: OTU

Excluded Variables[a]

Model		Beta In	t	Sig.	Partial Correlation	Tolerance	Collinearity Statistics VIF	Minimum Tolerance
1	SOC	-0.022[b]	-0.113	0.912	-0.029	0.622	1.607	0.622
	TN	0.137[b]	0.700	0.495	0.178	0.589	1.697	0.589
	TK	-0.076[b]	-0.486	0.634	-0.124	0.939	1.065	0.939
	TP	0.033[b]	0.218	0.830	0.056	0.994	1.006	0.994
	Cr	-0.011[b]	-0.067	0.948	-0.017	0.911	1.098	0.911
	Zn	0.153[b]	0.819	0.426	0.207	0.636	1.572	0.636
	Cu	0.154[b]	0.893	0.386	0.225	0.744	1.343	0.744
	Cd	0.063[b]	0.332	0.745	0.085	0.637	1.571	0.637
	Density	-0.092[b]	-0.400	0.695	-0.103	0.434	2.306	0.434
	pH	0.077[b]	0.504	0.621	0.129	0.988	1.013	0.988
	Pb	0.154[b]	1.012	0.327	0.253	0.944	1.059	0.944

a. Dependent Variable: OTU, b. Predictors: (Constant), WT.

Table S2. Multiple linear regression analyses between Ace and the tested environmental variables.

Variables Entered/Removed[a]

Model	Variables Entered	Variables Removed	Method
1	WT	.	Stepwise (Criteria: Probability-of-F-to-enter ≤ 0.050; Probability-of-F-to-remove ≥ 0.100).

a. Dependent Variable: Ace

Model Summary[b]

Model	R	R Square	Adjusted R Square	Std. Error of the Estimate	Durbin-Watson
1	0.729[a]	0.531	0.502	323.814	1.155

a. Predictors: (Constant), WT: b. Dependent Variable: Ace

ANOVA[a]

Model		Sum of Squares	df	Mean Square	F	Sig.
1	Regression	1901277.706	1	1901277.706	18.132	0.001[b]
	Residual	1677689.905	16	104855.619		
	Total	3578967.611	17			

a. Dependent Variable: Ace, b. Predictors: (Constant), WT.

Coefficients[a]

Model		Unstandardized Coefficients		Standardized Coefficients	t	Sig.	Collinearity Statistics	
		B	Std. Error	Beta			Tolerance	VIF
1	(Constant)	3906.864	168.062		23.247	0.000		
	WT	23.157	5.438	0.729	4.258	0.001	1.000	1.000

a. Dependent Variable: Ace

Excluded Variables[a]

Model		Beta In	t	Sig.	Partial Correlation	Tolerance	Collinearity Statistics VIF	Minimum Tolerance
1	SOC	0.028[b]	-1.858	0.083	-0.432	0.625	1.599	0.625
	TN	0.110[b]	-0.613	0.549	-0.156	0.167	5.985	0.167
	TK	-0.075[b]	1.778	0.096	.417	0.988	1.012	0.988
	TP	0.093[b]	-0.580	0.570	-0.148	0.390	2.562	0.390
	Cr	0.040[b]	0.522	0.609	0.134	0.959	1.043	0.959
	Zn	0.275[b]	-0.961	0.352	-0.241	0.990	1.010	0.990
	Cu	0.318[b]	0.751	0.464	0.190	0.433	2.309	0.433
	Cd	0.165[b]	0.473	0.643	0.121	0.952	1.050	0.952
	Density	-0.085[b]	1.325	0.205	0.324	0.839	1.192	0.839
	pH	0.158[b]	-0.603	0.555	-0.154	0.531	1.885	0.531
	Pb	0.271[b]	-0.697	0.497	-0.177	0.366	2.735	0.366

a. Dependent Variable: Ace, b. Predictors: (Constant), WT.

Table S3. Multiple linear regression analyses between Chao and the tested environmental variables.

Variables Entered/Removed[a]

Model	Variables Entered	Variables Removed	Method
1	WT	.	Stepwise (Criteria: Probability-of-F-to-enter ≤ 0.050; Probability-of-F-to-remove ≥ 0.100).

a. Dependent Variable: Chao

Model Summary[b]

Model	R	R Square	Adjusted R Square	Std. Error of the Estimate	Durbin-Watson
1	0.749[a]	0.561	0.533	307.209	1.256

a. Predictors: (Constant), WT: b. Dependent Variable: Chao

ANOVA[a]

Model		Sum of Squares	df	Mean Square	F	Sig.
1	Regression	1926778.108	1	1926778.108	20.440	0.000[b]
	Residual	1508272.836	16	94267.052		
	Total	3435050.944	17			

a. Dependent Variable: Chao, b. Predictors: (Constant), WT.

Coefficients[a]

Model		Unstandardized Coefficients		Standardized Coefficients	t	Sig.	Collinearity Statistics	
		B	Std. Error	Beta			Tolerance	VIF
1	(Constant)	3925.904	259.350		24.637	0.000		
	WT	23.312	5.156	0.749	4.521	0.000	1.000	1.000

a. Dependent Variable: Chao

Excluded Variables[a]

Model		Beta In	t	Sig.	Partial Correlation	Collinearity Statistics Tolerance	VIF	Minimum Tolerance
1	SOC	0.062[b]	0.286	0.779	0.074	0.622	1.607	0.622
	TN	0.137[b]	0.620	0.544	0.158	0.589	1.697	0.589
	TK	-0.047[b]	-0.264	0.795	-0.068	0.939	1.065	0.939
	TP	0.055[b]	0.322	0.752	0.083	0.994	1.006	0.994
	Cr	0.031[b]	0.173	0.865	0.045	0.911	1.098	0.911
	Zn	0.266[b]	1.308	0.210	0.320	0.636	1.572	0.636
	Cu	0.304[b]	1.666	0.116	0.395	0.744	1.343	0.744
	Cd	0.155[b]	0.737	0.473	0.187	0.637	1.571	0.637
	Density	-0.073[b]	-0.283	0.781	-0.073	0.434	2.306	0.434
	pH	0.157[b]	0.936	0.364	0.235	0.988	1.013	0.988
	Pb	0.258[b]	1.579	0.135	0.378	0.944	1.059	0.944

a. Dependent Variable: Chao, b. Predictors: (Constant), WT.

Table S4. Multiple linear regression analyses between Shannon and the tested environmental variables.

Variables Entered/Removed[a]

Model	Variables Entered	Variables Removed	Method
1	WT	.	Stepwise (Criteria: Probability-of-F-to-enter ≤ 0.050; Probability-of-F-to-remove ≥ 0.100).

a. Dependent Variable: Shannon

Model Summary[b]

Model	R	R Square	Adjusted R Square	Std. Error of the Estimate	Durbin-Watson
1	0.724[a]	0.524	0.494	0.202	1.609

a. Predictors: (Constant), WT: b. Dependent Variable: Shannon

ANOVA[a]

Model		Sum of Squares	df	Mean Square	F	Sig.
1	Regression	0.720	1	0.720	17.587	0.001[b]
	Residual	0.655	16	0.041		
	Total	1.374	17			

a. Dependent Variable: Shannon, b. Predictors: (Constant), WT.

Coefficients[a]

Model		Unstandardized Coefficients		Standardized Coefficients	t	Sig.	Collinearity Statistics	
		B	Std. Error	Beta			Tolerance	VIF
1	(Constant)	6.666	0.105		63.490	0.000		
	WT	0.014	0.003	0.724	4.194	0.001	1.000	1.000

a. Dependent Variable: Shannon

Excluded Variables[a]

Model		Beta In	t	Sig.	Partial Correlation	Collinearity Statistics		
						Tolerance	VIF	Minimum Tolerance
1	SOC	-0.159[b]	-0.717	0.485	-0.182	0.622	1.607	0.622
	TN	0.039[b]	0.169	0.868	0.044	0.589	1.697	0.589
	TK	0.137[b]	0.761	0.458	0.193	0.939	1.065	0.939
	TP	-0.322[b]	-2.039	0.060	-0.466	0.994	1.006	0.994
	Cr	-0.215[b]	-1.205	0.247	-0.297	0.911	1.098	0.911
	Zn	-0.136[b]	-0.617	0.546	-0.157	0.636	1.572	0.636
	Cu	-0.178[b]	-0.882	0.392	-0.222	0.744	1.343	0.744
	Cd	-0.212[b]	-0.977	0.344	-0.245	0.637	1.571	0.637
	Density	-0.152[b]	-0.569	0.578	-0.145	0.434	2.306	0.434
	pH	-0.030[b]	-0.167	0.870	-0.043	0.988	1.013	0.988
	Pb	-0.011[b]	-0.063	0.951	-0.016	0.944	1.059	0.944

a. Dependent Variable: Shannon, b. Predictors: (Constant), WT.

Table S5. Multiple linear regression analyses between the ratio of Proteobacteria and Acidobacteria (PA) and the tested environmental variables.

Variables Entered/Removed[a]

Model	Variables Entered	Variables Removed	Method
1	density		Stepwise (Criteria: Probability-of-F-to-enter ≤ 0.050; Probability-of-F-to-remove ≥ 0.100).
2	WT	.	Stepwise (Criteria: Probability-of-F-to-enter ≤ 0.050; Probability-of-F-to-remove ≥ 0.100).

a. Dependent Variable: PA

Model Summary[c]

Model	R	R Square	Adjusted R Square	Std. Error of the Estimate	Durbin-Watson
1	0.906[a]	0.821	0.810	0.542	
2	0.942[b]	0.888	0.873	0.443	2.808

a. Predictors: (Constant), density: Predictors: (Constant), density, WT; c. Dependent Variable: PA

ANOVA[a]

Model		Sum of Squares	df	Mean Square	F	Sig.
1	Regression	21.599	1	21.599	73.473	0.000[b]
	Residual	4.704	16	0.294		
	Total	26.303	17			
2	Regression	23.359	1	11.690	59.522	0.000[c]
	Residual	2.943	16	0.196		
	Total	26.303	17			

a. Dependent Variable: PA, b. Predictors: (Constant), density; c. Predictors: (Constant), density, WT.

Coefficients[a]

Model		Unstandardized Coefficients		Standardized Coefficients	t	Sig.	Collinearity Statistics	
		B	Std. Error	Beta			Tolerance	VIF
1	(Constant)	12.886	1.201		10.731	0.000		
	density	-5.917	0.690	-0.906	-8.572	0.000	1.000	1.000
2	(Constant)	10.479	1.268		8.262	0.000		
	density	-3.987	0.856	-0.611	-4.655	0.000	0.434	2.306
	WT	0.034	0.011	0.393	2.995	0.009	0.434	2.306

a. Dependent Variable: PA

Excluded Variables[a]

Model		Beta In	t	Sig.	Partial Correlation	Collinearity Statistics		
						Tolerance	VIF	Minimum Tolerance
1	SOC	0.210[b]	2.061	0.057	0.470	0.895	1.117	0.895
	TN	0.203[b]	1.872	0.081	0.435	0.818	1.223	0.818
	TK	-0.010[b]	-0.078	0.939	-0.020	0.720	1.390	0.720
	TP	0.034[b]	0.311	0.760	0.080	0.998	1.002	0.998
	Cr	0.178[b]	1.140	0.272	0.282	0.451	2.216	0.451
	Zn	-0.141[b]	-0.895	0.385	-0.225	0.459	2.187	0.459
	Cu	-0.072[b]	-0.531	0.603	-0.136	0.637	1.570	0.637
	Cd	-0.105[b]	-0.605	0.554	-0.154	0.388	2.578	0.388
	Pb	0.112[b]	1.007	0.330	0.252	0.902	1.108	0.902
	pH	-0.020[b]	-0.184	0.857	-0.047	0.997	1.003	0.997
	WT	0.393[b]	2.995	0.009	0.612	0.434	2.306	0.434
2	SOC	0.073[c]	0.630	0.539	0.166	0.578	1.730	0.280
	TN	0.070[c]	0.607	0.553	0.160	0.582	1.717	0.309
	TK	0.078[c]	0.728	0.479	0.191	0.667	1.500	0.308
	TP	0.052[c]	0.589	0.565	0.155	0.993	1.007	0.432
	Cr	-0.073[c]	-0.448	0.661	-0.119	0.296	3.376	0.141
	Zn	-0.099[c]	-0.763	0.458	-0.200	0.453	2.205	0.309
	Cu	-0.040[c]	-0.360	0.724	-0.096	0.631	1.586	0.367

Cd	-0.091[c]	-0.640	0.532	-0.169	0.387	2.581	0.264
Pb	0.113[c]	1.260	0.228	0.320	0.902	1.108	0.414
pH	0.042[c]	0.460	0.653	0.122	0.944	1.059	0.411

a. Dependent Variable: PA, b. Predictors: (Constant), density; c. Predictors: (Constant): density, WT
